# Supplementary material for: Whole Genome Sequencing of Avian Pathogenic Escherichia coli Causing Bacterial Chondronecrosis and Osteomyelitis in Australian Poultry
Source: Microorganisms. 2023 Jun 6;11(6):1513. doi: 10.3390/microorganisms11061513 (PMC10302171; doi:10.3390/microorganisms11061513)
Supplement: Supplementary file 1 [file microorganisms-11-01513-s001.zip › Figure Captions.pdf]

Figure S1: Pangenome-derived Core-genome phylogeny visualising relatedness of 205 $APEC_{BCO}$ strains and 95 $APEC_{colibac}$ strains. Tip points are coloured based on the collection of origin, while primary tip labels adjoined with dotted lines indicate strain names and are coloured based on sequence type. The next outermost band details the ST and e-serotype combinations, wherein the 10 most frequent STs are shown. Phylogroups for individual strains are denoted on the outermost lettered band adjacent to broad, coloured bars highlighting the general correspondence of clades and phylogroups. Tree is midpoint rooted.

Figure S2 – Box & Jitter plot visualising pairwise SNP distances (y-axis) between strains sharing a sequence type (x-axis). A) comparison between $APEC_{BCO}$ and APECcolibac collections; B) within $APEC_{colibac}$ collection, and C) within $APEC_{BCO}$ strains. A dotted red line (bottom) demarks a relatedness threshold of 100 SNPs.

Figure S3 – Virulence gene clustering of APEC genomes. This figure visualises non-metric multidimensional scaling clustering of genomes based on their carriage of virulence genes. Strains are coloured by: A) Collection of origin, or; B) Sequence type.

Figure S4 – Antimicrobial resistance gene clustering of APEC genomes. This figure visualises non-metric multidimensional scaling clustering of genomes based on their carriage of virulence genes. Strains are coloured by: A) Collection of origin;B) Sequence type, or; C) carriage of class 1 integrase *intI1*.

Table S1 – Strain and genome data. Details the metadata, genotypic data and biosample accession numbers for each genome under analysis.

Table S2 – IncF Replicon Sequence Types. Details the IncF Replicon Sequence Types (RSTs) among genomes under analysis

Table S3 – Genome wide association studies. This table details genes which were differentially abundant among $APEC_{BCO}$ and $APEC_{colibac}$ genomes under investigation, as per a Scoary-based pangenome wide association study.

Table S4 – Over and underrepresented genes amongst $APEC_{REP-BCO}$ genomes based on their sequence types implication in single or multi-site infections.
