# Supplementary figures and images for: Whole Genome Sequencing of Avian Pathogenic Escherichia coli Causing Bacterial Chondronecrosis and Osteomyelitis in Australian Poultry
Source: Microorganisms. 2023 Jun 6;11(6):1513. doi: 10.3390/microorganisms11061513 (PMC10302171; doi:10.3390/microorganisms11061513)

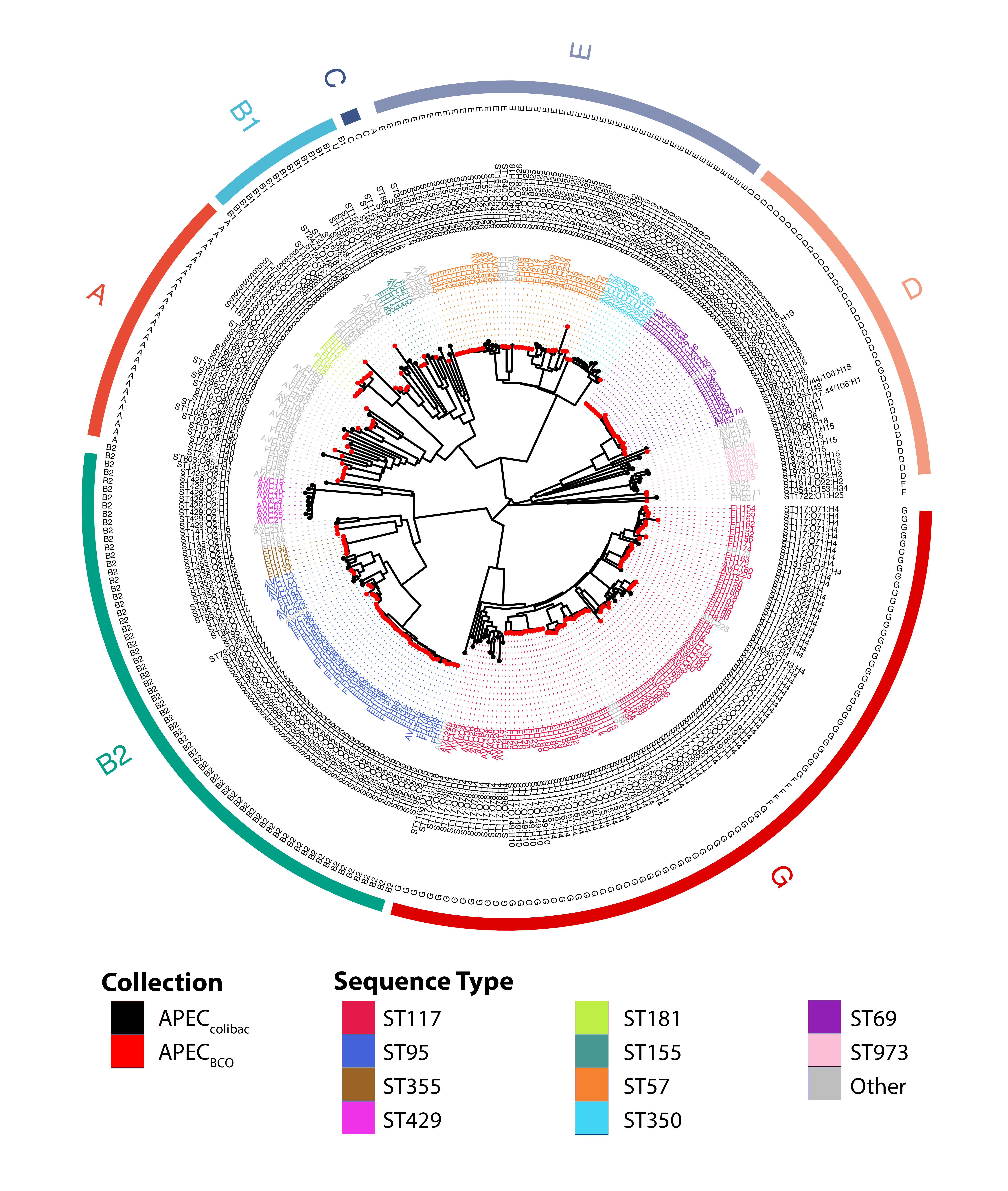

Supplement: Supplementary file 1 [file microorganisms-11-01513-s001.zip › Figure S1.png]

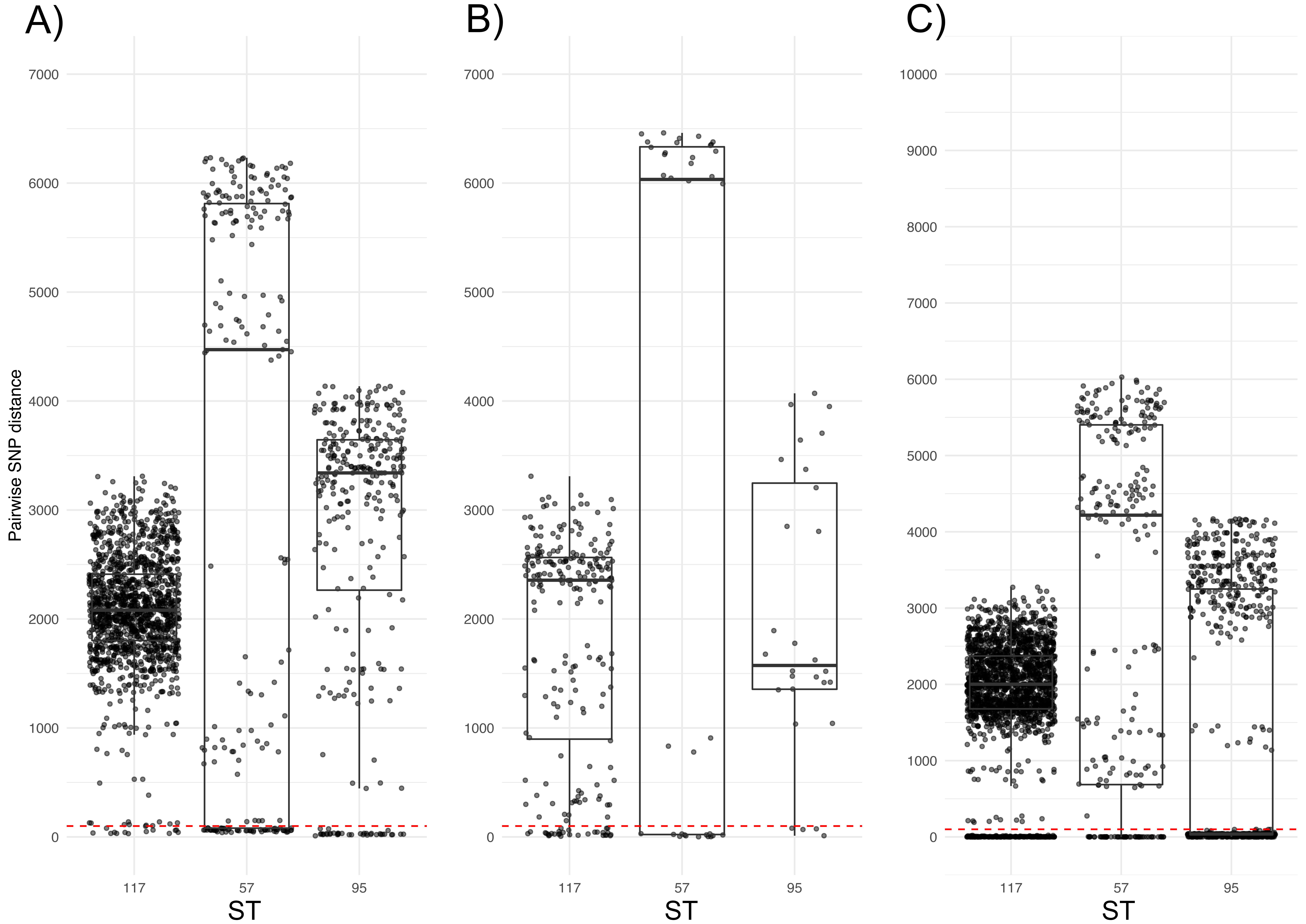

Supplement: Supplementary file 1 [file microorganisms-11-01513-s001.zip › Figure S2.png]

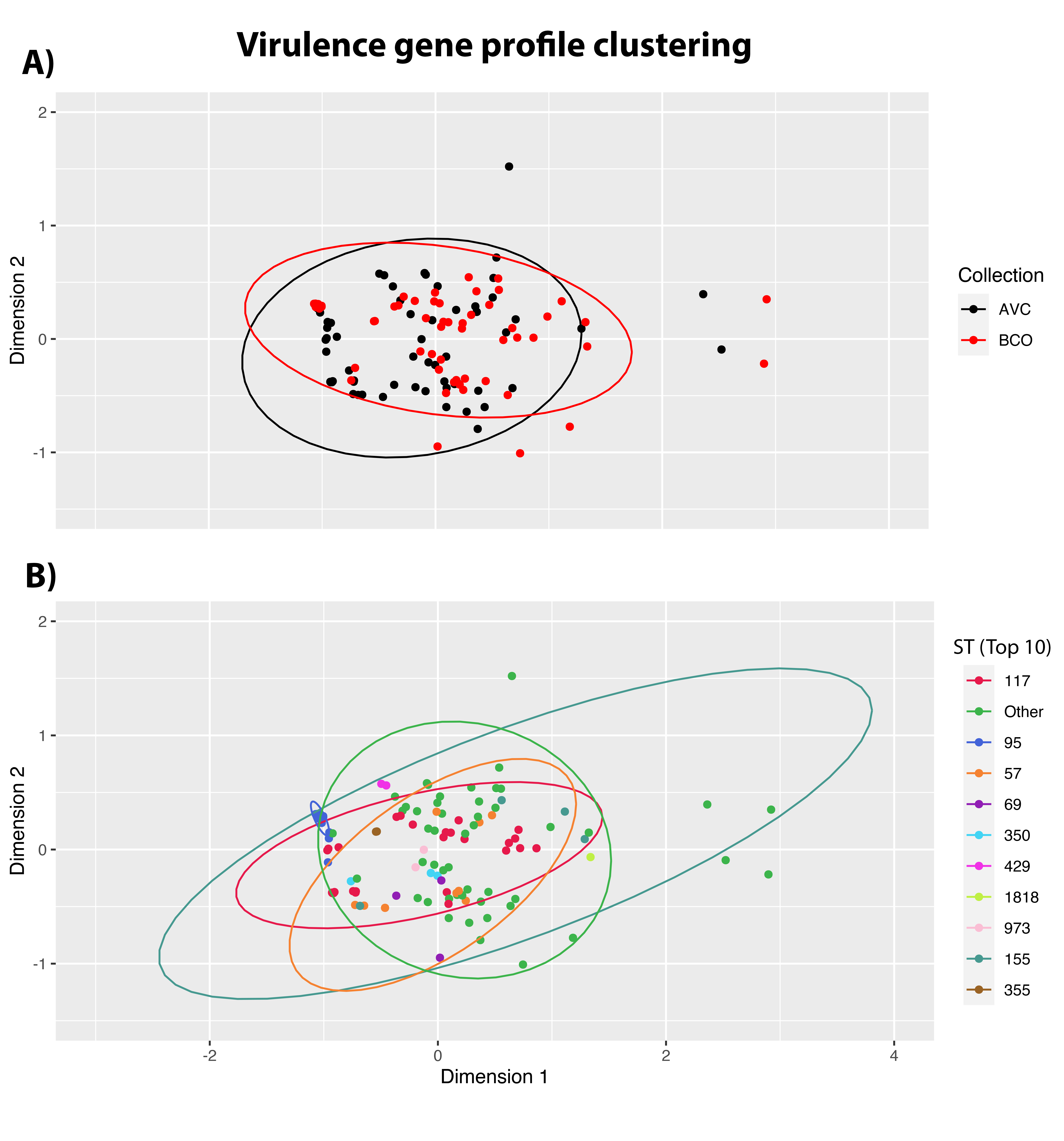

Supplement: Supplementary file 1 [file microorganisms-11-01513-s001.zip › Figure S3.png]

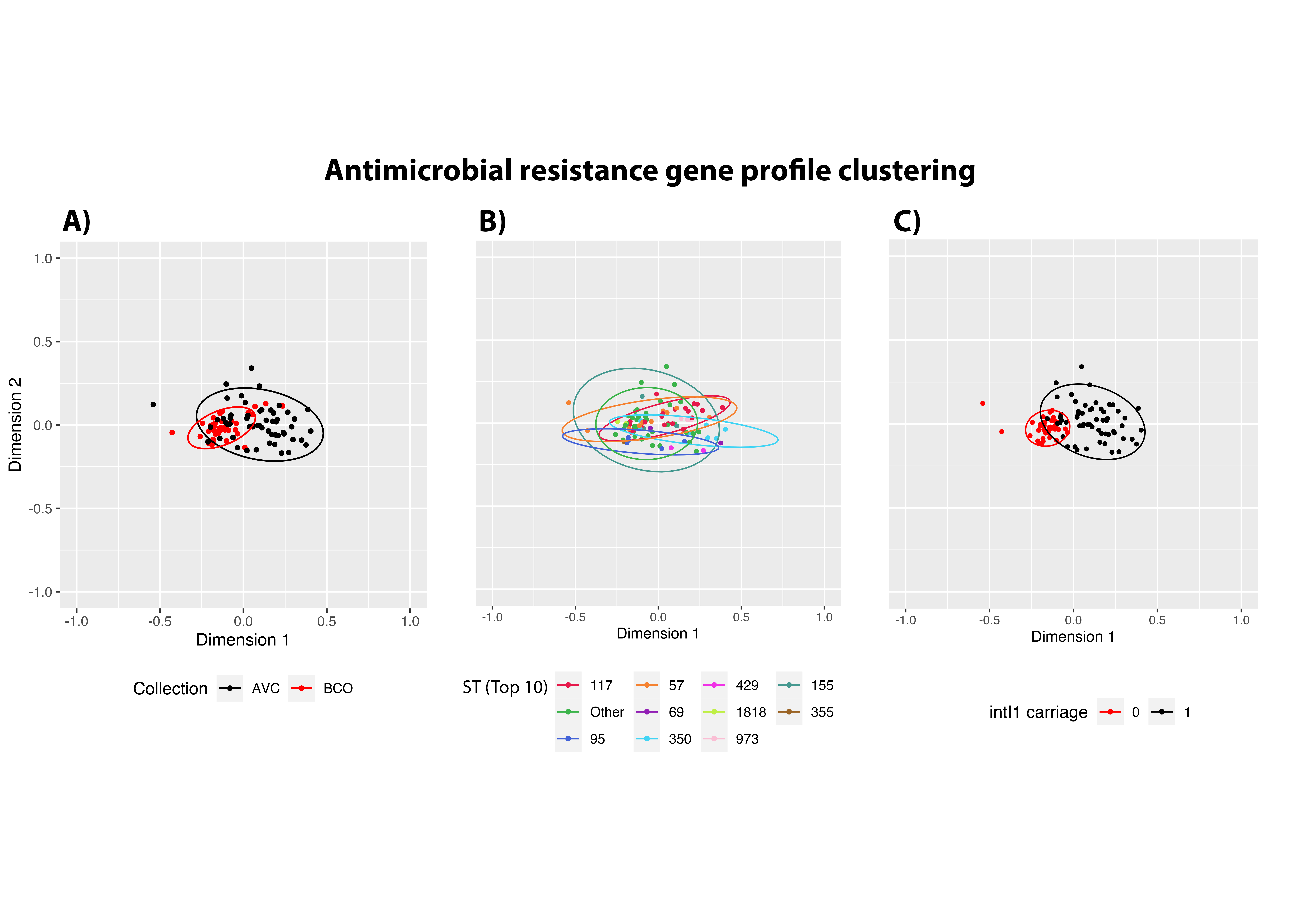

Supplement: Supplementary file 1 [file microorganisms-11-01513-s001.zip › Figure S4.png]
